# Mapping change in cycling infrastructure across Canada: What, where, and for whom?

**DOI:** 10.17269/s41997-025-01139-w

**Published:** 2025-12-11

**Authors:** Meghan Winters, Colin Ferster, Karen Laberee

**Affiliations:** Faculty of Health Sciences, Simon Fraser University, Burnaby, BC Canada

**Keywords:** Cycling, Built environment, Population groups, Public health surveillance, Equity, Cyclisme, Environnement bâti, Groupes de population, Surveillance de la santé publique, Équité

## Abstract

**Intervention:**

Governments are investing in safer cycling infrastructure to provide transportation options that support health, mobility, and environmental outcomes; these can be considered population health interventions.

**Research question:**

We aimed to measure change from 2022 to 2024 in cycling infrastructure in Canada to understand what and where changes happened, and who was impacted.

**Methods:**

We extracted data from OpenStreetMap.org (OSM) in 2022 and 2024 and coded them according to the Canadian Bikeway Comfort and Safety (Can-BICS) classification system (high, medium, or low comfort and safety). We measured differences (2022–2024) in length and type of cycling infrastructure within census subdivisions. We related differences in cycling infrastructure metrics with population composition within dissemination areas, nationally, by city size, and for particular cities, examining specific population groups (children, older adults, recent immigrants, racialized people, and low-income populations).

**Results:**

Total Can-BICS-OSM cycling infrastructure in 2024 increased to 27,098 km (15.3%) from 23,502 km in 2022. Most new infrastructure was multi-use paths (2725 km). High-comfort bike-only paths increased by nearly 50% (49 km). Nationally, access increased for recent immigrants, racialized people, and people with low incomes. Overall, areas with more children and older adults saw less increase in access to infrastructure, but within small and medium cities there were often increases.

**Conclusion:**

Using a national dataset, we detected an increase in cycling infrastructure in Canadian communities, with mobility and health implications for many equity-deserving population groups. The greatest increases, proportionally, in cycling infrastructure were seen in small- and medium-sized cities.

**Supplementary Information:**

The online version contains supplementary material available at https://doi.org/10.17269/s41997-025-01139-w.

## Résumé

**Intervention:**

Les gouvernements investissent dans des infrastructures cyclables plus sûres afin d'offrir des options de transport favorables à la santé, à la mobilité et à l'environnement; ces investissements peuvent être considérés comme des interventions en matière de santé publique.

**Question de recherche:**

Nous avons cherché à mesurer l'évolution des infrastructures cyclables au Canada entre 2022 et 2024 afin de comprendre quels changements ont eu lieu, où ils se sont produits et qui en a bénéficié.

**Méthodes:**

Nous avons extrait des données d'OpenStreetMap.org (OSM) en 2022 et 2024 et les avons codées selon le système de classification Canadian Bikeway Comfort and Safety (Can-BICS) (confort et sécurité élevés, moyens ou faibles). Nous avons mesuré les différences (2022 à 2024) en termes de longueur et de type d'infrastructures cyclables au sein des subdivisions de recensement. Nous avons mis en relation les différences dans les mesures des infrastructures cyclables avec la composition de la population dans les zones de diffusion, à l'échelle nationale, par taille de ville et pour des villes particulières, en examinant des groupes de population spécifiques (enfants, personnes âgées, immigrants récents, personnes racisées et personnes à faibles revenus).

**Résultats:**

Le réseau total d'infrastructures cyclables Can-BICS-OSM est passé de 23 502 km en 2022 (15,3%) à 27 098 km en 2024. La plupart des nouvelles infrastructures étaient des pistes polyvalentes (2 725 km). Les pistes cyclables en site propre et offrant un grand confort ont augmenté de près de 50% (49 km). À l'échelle nationale, l'accès s'est amélioré pour les immigrants récents, les personnes racisées et les personnes à faible revenu. Dans l'ensemble, les zones comptant davantage d'enfants et des personnes âgées ont connu une augmentation moins importante de l'accès aux infrastructures, mais dans les villes petites et moyennes, des augmentations ont souvent été observées.

**Conclusion:**

À partir d'un ensemble de données nationales, nous avons constaté une augmentation des infrastructures cyclables dans les collectivités canadiennes, ce qui a des répercussions sur la mobilité et la santé de nombreux groupes de population méritant l'équité. C'est dans les villes de petite et moyenne taille que l'on a observé la plus forte augmentation proportionnelle des infrastructures cyclables.

## Introduction

Transportation is an often-overlooked determinant of health, with the use of active modes such as walking, cycling, and rolling serving to reduce chronic disease risk and promote well-being (Prince et al., 2022; Rojas-Rueda et al., 2016). Safe infrastructure is critical to reducing the injury burden and allowing more people to choose to use active modes (Pucher & Buehler, 2016; Teschke et al., 2012). Motivated by a range of issues—from the climate emergency to providing access to opportunities to creating healthy built environments—Canadian governments (federal, provincial, municipal) have been investing in cycling infrastructure. These investments can be considered population health interventions—interventions made outside of the health sector that impact health and health equity (Hawe & Potvin, 2009; Mueller et al., 2015; Prince et al., 2025). However, cycling infrastructure investments have not been made equally across the country or even across cities, resulting in inequities in who has access to safe opportunities for cycling (Houde et al., 2018; Zhao et al., 2024). Evidence is needed on the scale of these changes, where they are happening, and who lives in the areas seeing change, so as to understand the potential impacts on population health and health equity.

Cycling research and practice has long been hampered by a lack of common nomenclature and consistent data sources that would enable national studies of investments in supportive environments for cycling. In 2020, the Canadian Bikeway Comfort and Safety (Can-BICS) classification system was created to provide a framework and common nomenclature that could be applied interjurisdictionally to datasets of cycling infrastructure (Winters et al., 2020). Numerous Canadian municipalities have spatial data for cycling infrastructure data available in open data portals; however, many communities—especially smaller ones—lack resources to release these or keep them up to date. Additionally, even within municipal datasets, there is a wide range of different terms for the same facilities, presenting a challenge for national or interjurisdictional comparisons (Winters & Zanotto, 2019). A response to this data challenge is OpenStreetMap.org (OSM), a volunteered map of the world that provides a consistent data source (Ferster et al., 2023). In 2022, algorithms were developed to apply the Can-BICS classification system to OSM data. This effort resulted in the creation of Can-BICS-OSM, the first publicly available, comprehensive, national network dataset of cycling infrastructure, classified by cycling comfort level (Ferster et al., 2023). These data were also used to create area-level metrics capturing the quantity and quality of cycling infrastructure for all dissemination areas in Canada, which allowed for linking to census data and spatial equity analyses of the cycling environments (Winters et al., 2022; Zhao et al., 2024). All of the data processes are reproducible, and a second dataset was released as a snapshot of the nation in 2024 (https://arcg.is/0G8yfX0).

Looking at cycling infrastructure investment as a population health intervention, it is important to be able to document where change happens in order to relate that to potential changes in health and social outcomes. In recent years, there have been documented boosts in cycling, especially in particular areas (Assunçao-Denis & Tomalty, 2019; Buehler & Pucher, 2021; MacEacheron et al., 2023). Through this time, in some cities, there has also been rapid implementation of new routes or upgrades of existing infrastructure (e.g. changing a painted bike lane to a cycle track) (Patterson, 2023; Victoria,  n.d.). There also have been (and continue to be) political motions calling for the removal of cycling infrastructure or restrictions on new construction (e.g., Canadian Press, 2025; MacDonald, 2025).

The national Can-BICS-OSM datasets offer research- and practice-ready metrics to track change over time and evaluate policy interventions. Furthermore, they provide a unique opportunity to take a truly national view of investments in cycling infrastructure across small, medium, and large Canadian cities, important given that contexts vary widely. In this paper, we aimed to measure change from 2022 to 2024 in cycling infrastructure in Canada. We look at change overall (nationally), by city size (small, medium, large), and for particular cities of interest. We also look at where changes have happened, and if this increases or decreases access to cycling infrastructure for equity-deserving population groups (specifically children, older adults, recent immigrants, racialized people, and people with low incomes). This work provides evidence on how recent changes in the built environment may be supporting health and health equity and may inform future investment strategies from different levels of government.

### Objective

We aimed to measure the change 2022 to 2024 in cycling infrastructure in Canada using Can-BICS-OSM to understand (1) what infrastructure changes happened, (2) where the changes happened, and (3) who was impacted by changes in terms of equitable access to bicycling infrastructure.

## Methods

### Data

To measure changes in Canada’s cycling infrastructure network, we used the Can-BICS-OSM network and metrics datasets for 2022 and 2024 (available at https://arcg.is/0G8yfX0). Can-BICS-OSM is a national cycling infrastructure dataset based on a validated classification of OpenStreetMap using consistent labels of comfort class and infrastructure type. High-comfort facilities are considered the lowest-stress facilities and include bike-only paths, cycle tracks, and local street bikeways. Medium-comfort bikeways are paved multi-use paths—considered comfortable for some people. Low-comfort bikeways are painted bike lanes—high-stress routes that are comfortable for few people. Within the Can-BICS classification, facility types that do not meet safety and comfort standards are classified as ‘non-conforming’. Most commonly, these are gravel paths, sharrows, or signed residential streets (Ferster et al., 2023). These non-conforming facilities (51,431 km in 2022 and 60,206 km in 2024) are not included in the current analysis. Rural areas tend to have very little infrastructure that meets the Can-BICS classification and do not meaningfully influence the results reported in this analysis.

Can-BICS-OSM area-level metrics are available for all Dissemination Areas (DAs) in Canada, as both continuous and categorical measures. We used the Can-BICS-OSM continuous metric, which reflects the cycling infrastructure within a 1-km buffer (excluding water) of the population-weighted centroid of the DA. The Can-BICS continuous metric sums the length of infrastructure using weighting by comfort class (3× high-comfort, 2× medium-comfort, and 1× low-comfort), which accounts for the different quality of infrastructure types (see Winters et al., 2022).

We used the Can-BICS-OSM area-level metrics that correspond with the 2021 Canada Census boundaries, since they are the most recent available. Note previous publications on the 2022 data used a version that aggregated based on the 2016 Canada Census boundaries and population, the most recent available at the time (e.g., Ferster et al., 2023; Winters et al., 2022; Zhao et al., 2024). We assumed that the population changes between the 2-year study interval were minimal.

## Analysis

To understand changes in the types of cycling infrastructure, we evaluated the change in the network dataset in the total length of infrastructure type, and by comfort class. As a convention for presentation in figures and tables, we show the totals in 2022 and 2024 and calculated the change as the length in 2024 minus the length in 2022.

To understand where the changes happened, we calculated the differences in length in the network dataset overall and by comfort class within Canada Census Subdivision (CSD) boundaries. CSD boundaries usually correspond with municipal boundaries, the jurisdictions where most cycling infrastructure is managed. CSDs cover 100% of the population in Canada. For our analysis, we stratified cities by population size: large cities with a population ≥ 500,000, medium cities with a population < 500,000 and  ≥ 50,000, small cities with a population < 50,000. We excluded CSDs with no population reported for the analysis by city size. There are 4830 CSDs that have populations reported. Of those, 737 have a population of 5000 or greater. The remaining 4093 CSDs have less than 5000 people.

To understand who was impacted by the changes and measure the spatial equity of access, we evaluated how changes in the Can-BICS continuous metric related to the population composition. We followed methods developed by Zhao et al. 

(2024), using the population data from the 2021 Statistics Canada Census of Population for the following equity-deserving population groups: children ages 14 years or younger, older adults 65 years of age or older, recent immigrants (moved to Canada within the last 10 years), racialized people (based on the Statistics Canada label of visible minority), and people with low incomes (based on the Low-Income Measure After Tax (LIM-AT)). We correlated the differences in the Can-BICS continuous metric with the composition of the population (i.e., proportion of the total population for each equity-deserving population group) overall, by city size, and for particular cities of interest. Cities of interest were selected to represent a range of geographies.

## Results

### What types of changes happened

The total length of cycling infrastructure in the Can-BICS-OSM 2022 dataset was 23,502 km, and in 2024 it was 27,098 km, representing a 15.3% increase in the length of the Can-BICS network (Table 1). We found that cycling infrastructure length increased for all Can-BICS comfort classes and infrastructure types. By length, most of the increase was for multi-use paths (which are medium comfort) (2725 km, 75.8% of the total change). Proportionally, bike-only paths (high comfort) increased the most (49 km, a 46.7% increase in length). While painted lanes (low comfort) increased the second most by length (492 km), this was the smallest proportional increase (a 5.8% increase).

**Table 1:** National inventory of cycling infrastructure by Can-BICS^1^ comfort class and infrastructure type using OSM^2^ data from all CSDs^3^ (inspired by Table 5 in Ferster et al., 2023). For the difference from 2022 to 2024, % change is the percentage of total change for each infrastructure type, while % pt is the percentage points change of the composition of each infrastructure type

Comfort class	Infrastructure type	2022		2024		Change			
		km	%	km	%	km	%	% of total change	% pt
High	Bike-only path	105	0.4	154	0.6	49	46.7	1.4	0.1
	Cycle track	1830	7.8	2021	7.5	191	10.4	5.3	−0.3
	Local-street bikeway	1374	5.8	1513	5.6	139	10.1	3.9	−0.3
Medium	Multi-use path	11,678	49.7	14,403	53.2	2725	23.3	75.8	3.5
Low	Painted lane	8515	36.2	9007	33.2	492	5.8	13.7	−3
**Total**	**Total OSM-Can-BICS**	23,502	100	27,098	100	3596	15.3	100	0

^1^*Can-BICS* Canadian Bikeway Comfort and Safety classification system

^2^*OSM* OpenStreetMap.org

^3^*CSD* Census subdivision; an administrative boundary used by Statistics Canada that generally reflects municipalities or areas treated as municipal equivalents

### Where did the changes happen?

**Fig. 1:**
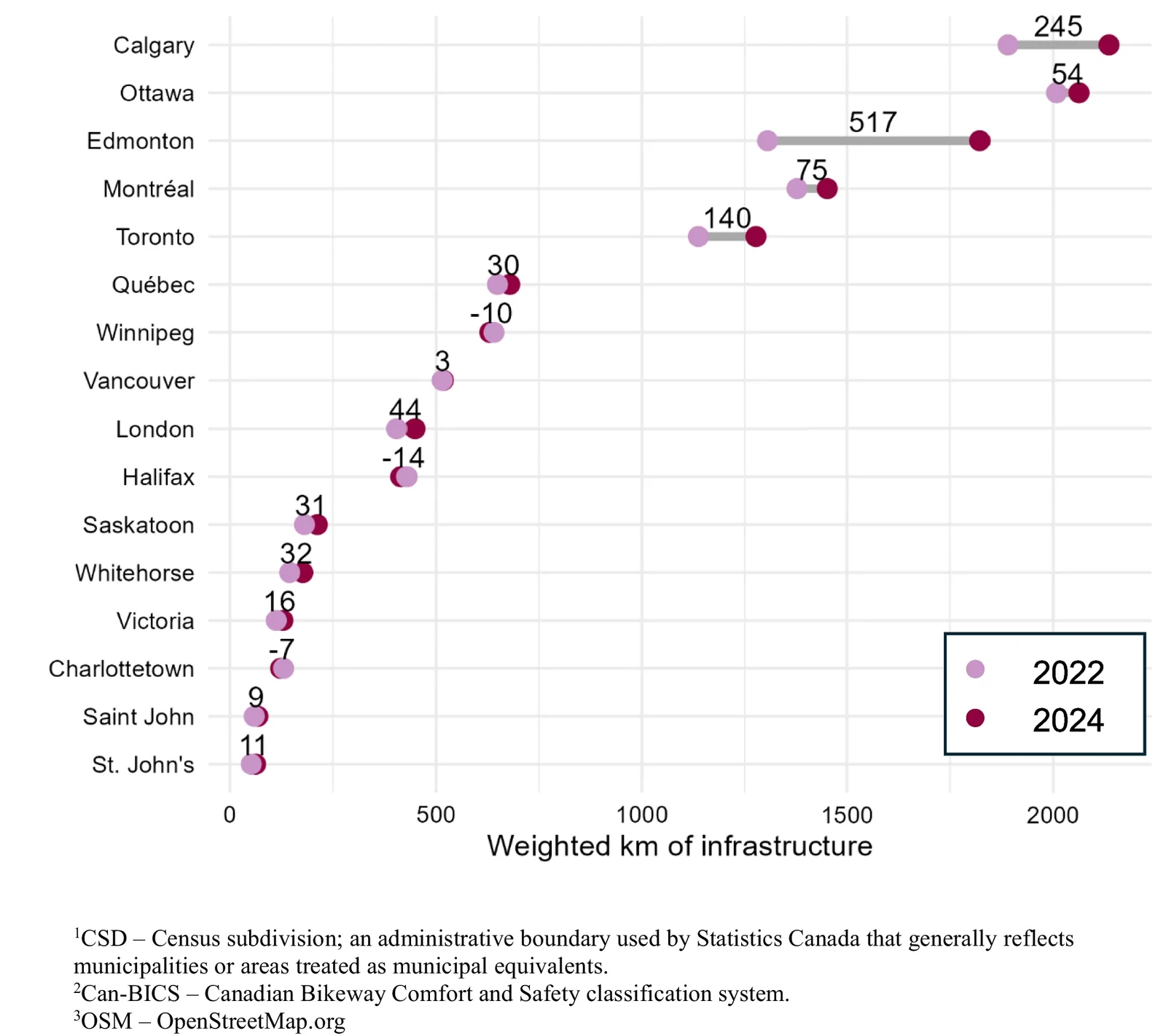
Change in infrastructure for selected cities (CSDs^1^), using weighted length of Can-BICS^2^ infrastructure (high comfort × 3, medium comfort × 2, and low comfort × 1). The numbers above the lines show the difference in weighted infrastructure length between years (where positive numbers indicate an increase). Data obtained from OSM^3^ in January 2022 and 2024

**Fig. 2:**
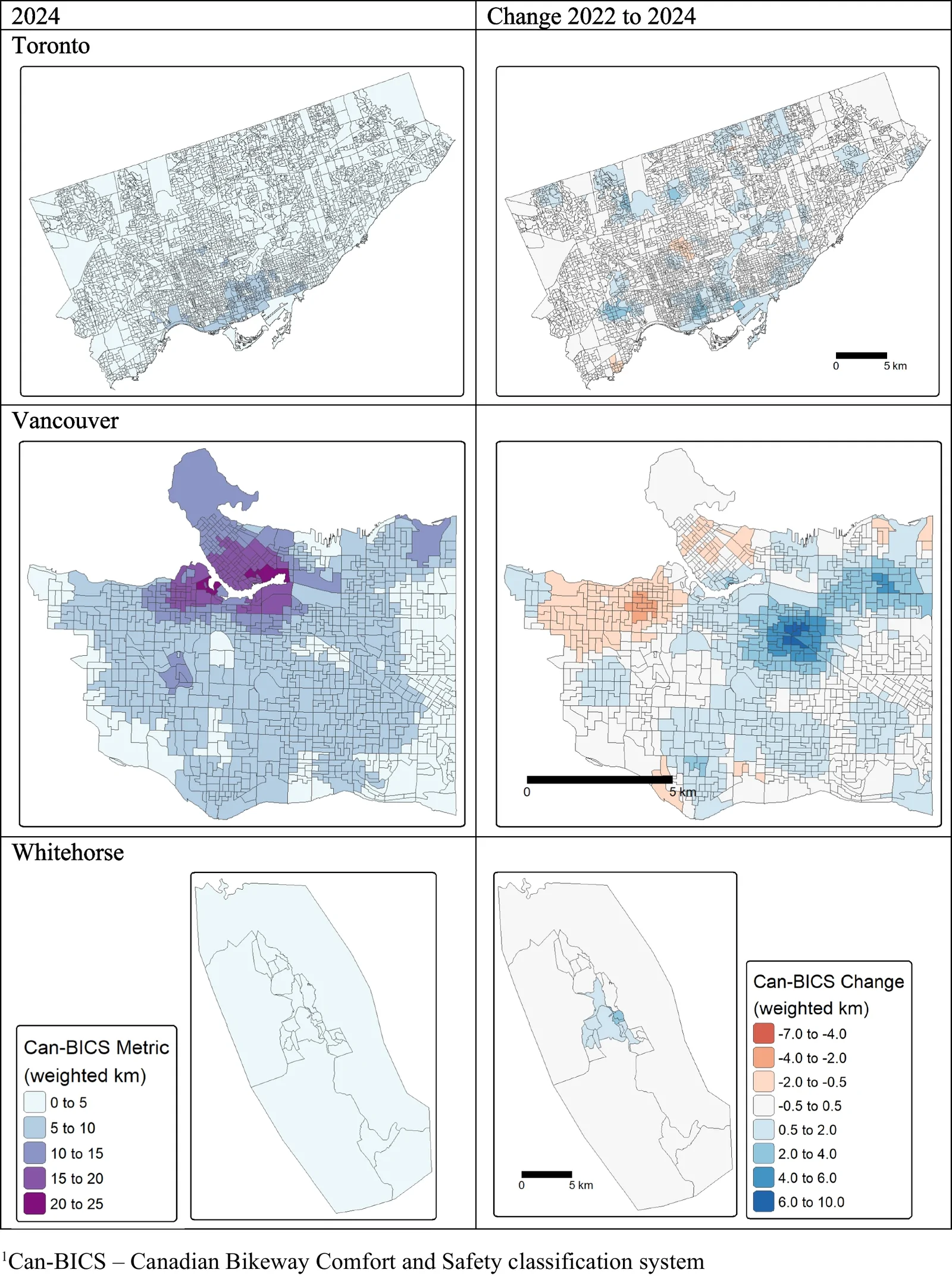
Can-BICS^1^ continuous metric for 2024 and change in selected cities from 2022. Data from OSM^2^

Comparing the changes by city size, notably, more Can-BICS infrastructure was built in small and medium cities than in large cities (1647 km or 16.5% increase in small cities, 1206 km or 16.1% increase in medium cities, and 742 km or 12.3% increase in large cities) (Table 2). Considering specific cities, some large cities with established cycling networks (such as Vancouver and Ottawa) showed little change over the time period, while some growing prairie cities (such as Edmonton and Calgary) and small and medium cities had extensive Can-BICS infrastructure networks and high rates of growth (Fig. 1). We mapped the Can-BICS-OSM continuous metric in 2024, and the change from 2022 to 2024, to look at patterns of change within cities (Toronto, Vancouver, and Whitehorse shown in Fig. 2). These figures visualize the spatial patterning of where changes in infrastructure happened. A supplementary table of the changes observed in all 737 communities with a population greater than 5000 can be accessed through this link: https://summit.sfu.ca/item/39991.

**Table 2:** National inventory of cycling infrastructure from OSM^1^ by Can-BICS^2^ comfort class within CSDs^3^, by city size. Large cities have population ≥ 500,000, medium cities have population < 500,000 and ≥ 50,000, and small cities have population < 50,000. Only CSDs with populations reported are included.

Size	Can-BICS infrastructure		2022		2024		Change			
	Comfort class	Type	km	%	km	%	km	%	% of total change in city size class	% pt
Large cities	High	Bike-only path	61	1	84	1.2	23	37.7	3.1	0.2
		Cycle track	538	8.9	701	10.3	163	30.3	22	1.4
		Local street bikeway	654	10.8	666	9.8	12	1.8	1.6	−1
	Medium	Multi-use path	3048	50.5	3544	52.2	496	16.3	66.8	1.8
	Low	Painted bike lane	1740	28.8	1788	26.4	48	2.8	6.5	−2.4
	**Total**	**Total OSM-Can-BICS**	6041	100.0	6783	100.0	742	12.3	100.0	0
Medium cities	High	Bike-only path	21	0.3	34	0.4	13	61.9	1.1	0.1
		Cycle track	608	8.1	724	8.3	116	19.1	9.6	0.2
		Local street bikeway	334	4.5	392	4.5	58	17.4	4.8	0
	Medium	Multi-use path	3263	43.6	3965	45.6	702	21.5	58.2	2
	Low	Painted bike lane	3265	43.6	3582	41.2	317	9.7	26.3	−2.4
	**Total**	**Total OSM-Can-BICS**	7491	100.0	8697	100.0	1206	16.1	100.0	−0.1
Small cities	High	Bike-only path	23	0.2	35	0.3	12	52.2	0.7	0.1
		Cycle track	682	6.9	595	5.1	−87	−12.8	−5.3	−1.7
		Local street bikeway	386	3.9	455	3.9	69	17.9	4.2	0
	Medium	Multi-use path	5355	53.8	6882	59.3	1527	28.5	92.7	5.5
	Low	Painted bike lane	3508	35.2	3634	31.3	126	3.6	7.7	−3.9
	**Total**	**Total OSM-Can-BICS**	9954	100.0	11,601	100.0	1647	16.5	100.0	0

^1^*OSM* OpenStreetMap.org

^2^*Can-BICS* Canadian Bikeway Comfort and Safety classification system

^3^*CSD* Census subdivision; an administrative boundary used by Statistics Canada that generally reflects municipalities or areas treated as municipal equivalents

### Who was impacted by the changes?

**Fig. 3:**
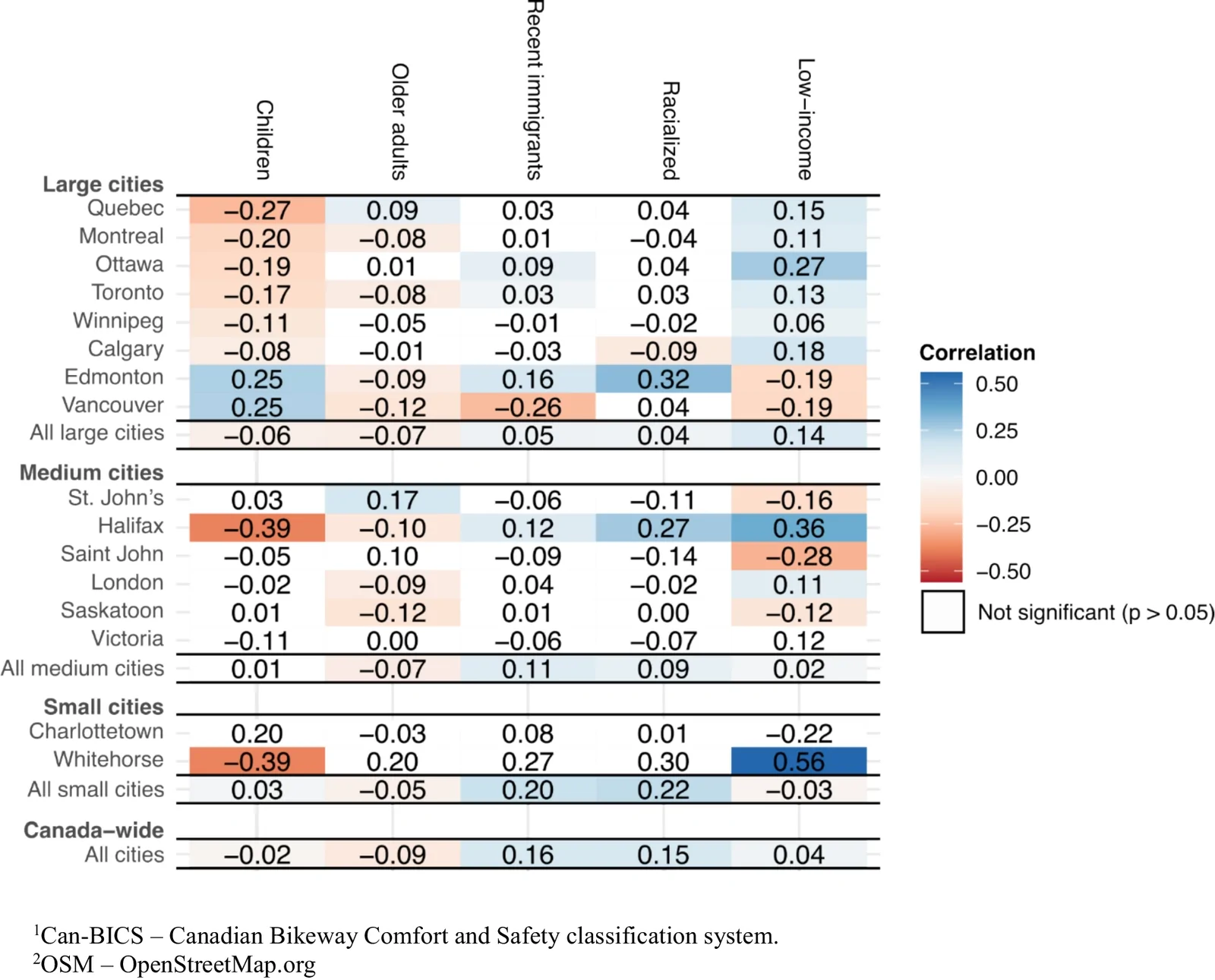
Pearson correlation coefficients between the change from 2022 to 2024 in the Can-BICS^1^ continuous metric and the composition of the population for population groups (proportion of the population). Significant values (*p* ≤ 0.05) are filled (blue for positive and red for negative associations). Cities are grouped by population and ordered from east to west. Data from OSM^2^. Inspired by Table 2 in Zhao et al. (2024)

The changes measured showed that at the national level, cycling infrastructure additions (2022 to 2024) generally tended to go into areas with lower proportions of populations of children and older adults, and more cycling infrastructure additions happened in areas with greater populations of recent immigrants, racialized people, and people with low incomes. Figure 3 shows Pearson correlation coefficients Canada-wide, by city size, and for a subset of cities within each of those categories. Statistically significant correlations are in colour, and the magnitude of the correlation is visualized by the intensity of shading. Blue indicates positive trends (new infrastructure in areas with higher proportions of the equity-deserving population), while red indicates negative associations (no/less new infrastructure in areas with higher proportions of that equity-deserving population). Figure 3 shows generalized aggregate trends for all cities across Canada (see lines ‘all large cities’, ‘all medium cities’, ‘all small cities’). On average across all large cities (‘all large cities’ line), we see significant positive correlations with areas with greater populations of recent immigrants, racialized people, and people with low incomes, and negative correlations for areas with more children or more older adults. The table also shows select city-specific results. The disaggregate data for select cities show patterns that hold for many large cities but are inverse in others (e.g. Edmonton, Vancouver). In the medium and smaller cities overall (‘all medium cities’ and ‘all small cities’), we see similar patterns, albeit smaller in magnitude, and trends for areas with more children were not significant. In terms of specific cities, from the set included here, Whitehorse and Halifax stand out in terms of stronger correlations, with greater change in areas with more low-income populations and lesser change in areas with more children.

## Discussion

In this work we leverage the first consistently coded Canadian dataset on cycling infrastructure to examine change over time. The availability of such a resource enables large-scale analyses of spatial patterns where cycling is supported, where investment is happening, and which areas may be overlooked. Most cycling infrastructure projects are managed by municipalities, and as a result, datasets and prioritization initiatives are often geographically fragmented. The Can-BICS-OSM dataset is built from the volunteered global OSM dataset (OpenStreetMap Contributors, 2024), enabling a single source of national data on cycling infrastructure. Previous work evaluating the OSM dataset has shown that it identifies cycling infrastructure as well as municipal datasets, and that may often be more up to date (Ferster et al., 2019). Leveraging open data sources like OSM is essential to understanding the state of the world of cycling infrastructure and mapping change across larger geographies. Using this rich data resource, in this paper, we are able to look at what types of changes are happening, where they are happening, and who is impacted.

Monitoring the availability of infrastructure that provides safe, comfortable opportunities for cycling is crucial for public health and planning practice. Implementing high-quality cycling infrastructure is a health-promoting built environment intervention that encourages people to be physically active, provides access to opportunities, and reduces exposure to the risk of traffic injury. The current study found a 15.3% increase in the length of cycling infrastructure across Canada over 2022 to 2024—a substantial increase in scale, the vast majority of this distance being multi-use paths. Changes picked up in OSM may represent newly built routes, or they may represent upgrades of existing routes that did not previously meet Can-BICS classifications (e.g., paving gravel paths; adding traffic calming and traffic diversion measures that would convert residential routes to the standard of ‘local street bikeways’). Within the Can-BICS infrastructure types, the dataset can also capture where cities are increasing the quality of infrastructure, for example, as they upgrade from painted bike lanes (low-comfort facilities, under Can-BICS classification) to cycle tracks (high comfort). These higher comfort facilities are instrumental in encouraging people to try cycling or to cycle more often (Hull & O’Holleran, 2014; Pearson et al., 2023). Tracking changes over time provides important long-term data on how well Canada is supporting healthy transportation options in cities of all sizes. Unfortunately, this is of particular relevance given that, as we see in Ontario and elsewhere, cycling infrastructure can also be removed for political arguments, despite it being the public health evidence base (Harrison, 2025).

Practitioners consider a range of factors in deciding where to implement new infrastructure, be it cost, public acceptability, land use, network gaps, opportunities related to development or public works—and also social equity. Understanding where changes to cycling infrastructure are being made helps identify who stands to benefit or be left behind. With an eye to distributional justice, social equity analyses allow practitioners to evaluate how cycling infrastructure investments are allocated. Our analyses of the 2022 Can-BICS-OSM dataset showed that not all population groups in Canada have equal access to cycling infrastructure (Zhao et al., 2024). The existing urban form and cycling infrastructure patterns in Canadian cities reflect historical political decisions and settlement and development patterns. A common pattern in many cities’ cycling infrastructure is a focus on connecting to business districts, but often fewer connections between neighbourhoods. According to Lam (2022), this ‘radial’ approach to planning (prioritizing journeys from urban peripheries into city centres during peak hours) reflects a male bias in transportation planning. In contrast, a more intersectional perspective would support an ‘orbital’ approach to cycling routes, such as connecting neighbourhoods, which enables more cycling for diverse purposes (e.g. care-related trips). A focus on social equity can look at spatial access in terms of who has safe infrastructure to cycle from their home to the destinations they need to reach. Applying equity-driven approaches for planning future investments, city staff may evaluate the existing cycling network and prioritize investments for populations who have been underserved or are underrepresented in the cycling community (Williams et al., 2023). In the current analysis, we see that Canada-wide, cycling infrastructure investments from 2022 to 2024 have continued to be in areas with more recent immigrants, racialized people, and people with low incomes, and not in areas with more children or more older adults. Such investments may continue to exacerbate some of the existing inequities seen for younger and older populations (Zhao et al., 2024).

Infrastructure metrics (and measuring the changes in them) are influenced by the underlying urban form and nature of the administrative boundaries of each city. Careful contextual interpretation is needed in making comparisons between and within cities. Large, agglomerated, or less densely populated cities such as Toronto, Edmonton, and Calgary contrast with cities that are smaller geographically/nested within a region, such as Victoria and Vancouver. Cities with larger geographic areas tend to have greater lengths of cycling infrastructure due to their size; they often include long distances of multi-use paths and painted bike lanes through more rural areas. To account for these contextual factors, we explored normalization strategies including area and population size. We found that normalizing by area (e.g. km infrastructure/land area) emphasized changes in the most concentrated/least sprawling cities. Normalizing by population (e.g. km infrastructure/population) emphasized changes in small cities—especially those that serve non-permanent populations, such as tourists or military personnel. Normalizing by population can also make the change estimates very sensitive to small changes in cities with small populations. In the current study, we report on total distance (not normalized). We caution readers to consider the impact of differences in urban form and administrative boundaries in considering which metrics are most suitable for research or local decision-making.

OSM data are improving over time both as the rates of mapping increase and as ongoing editing corrects mistakes in mapped features. This is progress; however, it can also be a challenge to discern if observed changes arise from improvements in mapping and tagging or reflect real infrastructure changes on the ground. Despite the needs of built environment researchers, measuring change is outside of best practices on OSM; rather, OSM’s mandate is only to show what is currently visible on the ground (OpenStreetMap Wiki, 2025). Based on spot checks, we surmise that the amount of error is small and, in many cases, random (unbiased). One situation where there may be systematic over-estimation of change is where previously missing infrastructure is added to the map; this effect could be largest in small and medium cities with growing populations, where mapping was previously incomplete. Still, improvements to the data on OSM are always welcome despite the challenges they present in monitoring changes over time.

Beyond OSM, there are other efforts underway to develop national inventories of cycling infrastructure: examples include compilations of municipal data (led by Statistics Canada, 2025) and machine learning approaches using Google Streetview data (led by the Public Health Agency of Canada, amongst others). Each approach offers strengths and limitations. For example, while municipal data tends to be consistently attributed within a city, it may not be consistent in year of implementation or nomenclature terminology between different cities (Winters & Zanotto, 2019). Collating municipal data from multiple sources requires considerable labour. Municipal data is not publicly available in all communities in Canada, making it impossible to create a wall-to-wall map, and it may be the smaller and less-resourced communities where data is missing. Google Streetview has been useful for reliable spot checks, but many cities may have infrequent captures (for example, Whitehorse was captured in 2009 and 2022). Ultimately, as the diverse methodologies advance, they provide opportunities to cross-check, validate, and potentially combine data, and to advance cycling research and practice in Canada.

There are limitations to this work. Our methodology relies on volunteer-mapped data that may be incomplete but is only improving over time. We looked at equity only through a distributional lens, not procedural or recognitional equity considerations (Meerow et al., 2019). Our work captures the presence of infrastructure, but an outstanding data gap is around how well it is used. Finally, especially in smaller communities, people may use unpaved or informal routes for cycling, but these types of infrastructure are not included in our analysis. Better capturing these facilities that are important connections in rural and remote areas is an area of our ongoing work.

## Conclusions

Cycling is a healthy, convenient transportation mode that is best supported by providing safe, comfortable infrastructure. Over decades, the investments in cycling infrastructure have not been made consistently across Canada or even across jurisdictions. Monitoring where changes in infrastructure are happening and who is impacted is necessary for national public health surveillance of the built environment. With this work, we demonstrate how to monitor change over time in cycling infrastructure on a national scale. Using these national Can-BICS-OSM datasets, we can examine investments across contexts and identify who may be left behind and where. This methodology can be used again at future timepoints, offering the potential to explore linkages between cycling infrastructure and health and social outcomes.

## Contributions to knowledge

What does this study add to existing knowledge?

- We measured increases in cycling infrastructure nationally from 2022 to 2024.
- Canadian cities are investing in medium- and high-comfort cycling facilities.
- Open datasets such as OSM provide information on cycling infrastructure in all areas of Canada, albeit with some challenges in discerning change in the built environment from improved mapping accuracy.

What are the key implications for public health interventions, practice, or policy?

- Monitoring national change in cycling infrastructure is important for public health surveillance of built environments across all jurisdictions.
- Investments in high-quality cycling infrastructure are important to support safe active travel, especially for underserved populations.
- Changes in cycling infrastructure may reflect increased investment or better mapping, or both.

## Supplementary Information

Below is the link to the electronic supplementary material.ESM 1Supplementary Material 1: Also available here: https://summit.sfu.ca/item/39991. (XLSX 55.8 KB)

## Data Availability

The dataset is available here: https://www.arcgis.com/home/item.html?id=efaf04c6e3914c059bfb7298e784a89c

## Code availability

https://github.com/streckereck/osm_can_bics
